# Supplementary material for: Learning curve and surgical outcome of robotic assisted colorectal surgery with ERAS program
Source: Sci Rep. 2022 Nov 29;12:20566. doi: 10.1038/s41598-022-24665-w (PMC9709162; doi:10.1038/s41598-022-24665-w)
Supplement: Supplementary file 2 — Supplementary Information 2. [file 41598_2022_24665_MOESM2_ESM.zip › RAL Raw data-Ñ[▒K/068/HS2_ñw╝╨░O▒Kñσ.pdf]

正式摘要(電子簽章：000000219845109)

W72 / W72 ☐ 英文標題(注意:無英文姓名-請補病人基本檔!) ☐【讀取】憑證

臺中榮民總醫院

出院病歷摘要

轉入號 : 轉出號 :

[redacted]

**(8)**流水編號	
**(9)**入院日期	110/06/01 (2021) 直腸外科 W72 - 105
**(10)**轉科日期	
**(11)**出院日期	110/06/30 (2021) 住院天數計 29 日

診斷

- 入院 :[A/P]
  s/p LAR with dehescience and abscess formation over anastomosis site
  [Hx]
  1. Rectum adenocarcinoma, 5cm FAV, cT3N1bM0, Stage IIIA, ypT0N0
  CCRT(54Gy/30frs, 5-FU+LV 2020/12/14, 12/21, 2021/1/4, 1/18, 1/25, 2/9), with anal skin erosion s/p EGF 2021/2/1, s/p FOLFOX 2021/2/23, 3/9
  s/p robotic LAR + loop ileostomy on 2021/3/31
  2. BPH s/p green light laser on 2021/2/5
  3. A 3.0mm ground glass nodule at RLL, r/o lung cancer
- 出院 :[A/P]
  s/p LAR with dehescience and abscess formation over anastomosis site, s/p PCD insertion(presacral)on 2021/06/04, s/p redo Low anterior resection with colo-anal anastomosis + Ileostomy on 2021/06/16.
  [Hx]
  1. Rectum adenocarcinoma, 5cm FAV, cT3N1bM0, Stage IIIA, ypT0N0
  CCRT(60Gy/30frs, 5-FU+LV 2020/12/14, 12/21, 2021/1/4, 1/18, 1/25, 2/9), with anal skin erosion s/p EGF 2021/2/1, s/p FOLFOX 2021/2/23, 3/9
  s/p robotic LAR + loop ileostomy on 2021/3/31
  2. BPH s/p green light laser on 2021/2/5
  3. A 3.0mm ground glass nodule at RLL, r/o lung cancer

主訴

- Rectal pain for two months.

病史

- The 66-year-old male had history of
  1. Rectum adenocarcinoma, 5cm FAV, cT3N1bM0, Stage IIIA, ypT0N0
  CCRT(54Gy/30frs, 5-FU+LV 2020/12/14, 12/21, 2021/1/4, 1/18, 1/25, 2/9), with anal skin erosion s/p EGF 2021/2/1, s/p FOLFOX 2021/2/23, 3/9
  s/p robotic LAR + loop ileostomy on 2021/3/31
  2. BPH s/p green light laser on 2021/2/5
  3. A 3.0mm ground glass nodule at RLL, r/o lung cancer

  The patient was just hospitalized during 2021/03/30-04/07 for robotic-assisted LAR. Surgical pathology revealed no residual tumor. He was then under regular CRS OPD for help. Due to persistent rectal pain was noted, abdominal CT was arranged on 04/16, showing infiltrative soft tissue around the rectal anastomosis site. Pain got improving after analgesic use but it didn't subside. Due to the symptoms got more severe, he went to our ER for help on 05/31. Lab had no remarkable findings. No fever nor rebounding pain were found. Abdominal CT on 05/31 revealed R/O abdominal abscess over anastomosis site.
  This time, under the impression of s/p robotic-assisted LAR with suspect abscess formation over rectal anastomosis site, the patient was admitted for CT-guided drainage.

體檢發現

- CEA: 2.8(2020/12)--2.8(2021/1)
  2020/11 CT: no mets.

- 2020/12 PET: uptake at the rectum (SUVmax:9.4/1h)
  2021/1 MRI: rectum ca. Lung CT: RLL mass.
  21/4 minor leak in anas.
  - General appearance :
    - easy looking , conscious clear , without cardiopulmonary distress
    - Vital signs自動帶入date 體溫(BT) 脈搏(P) 呼吸(R) 血壓(BP) 身高(BH) 體重(BW) BMI
      20210601 36.8C 75/min 18/min 133/75mmHg
    - Vital signs: B.P. 133/75 mmHg , P.R. 75 /min , R.R. 18 /min , B.T. 36.8 ℃
  - HEENT :
    - Eyes: no anemic conjunctiva, no icteric sclera, normal EOM
    - ENT: no otorrhea, no rhinorrhea, no erythematous mucosa, no injected tonsil, no deformity
  - Neck: supple, freely movable, trachea midline,no deformity, no engorged jugular vein , no palpable thyroid or lymph node enlargement
  - Chest:
    - Inspection: Grossly normal, symmetric and fully expansion, no accessory muscle used, no operation scar, no spider angiomata, no gynecomastia
    - Palpation: no palpable mass or nodule, no subcutaneous emphysema, no tenderness
    - Ausculation: clear breathing sound
  - Heart:
    - Percussion: normal heart size
    - Ausculation: regular heart beat, normal S1, S2, no gallop, no murmur
  - Abdomen:
    - Inspection: in
      - flat contour, symetrically, no scar, no stria, no dilated veins, no rashes
    - Ausculation: normoactive bowel sound, no abdominal bruits
    - palpation: soft, no muscle guarding or rigidity, no local tenderness, no rebounding pain, no palpable mass, no palpable pulsation
    - Percussion: no shifting dulllness, no hepatomegaly, no splenomagaly
  -

Peripheral pulsation :	CA	BA	RA	FA	PA	PTA	DPA
Right side :	++	++	++	++	++	++	++/+++
Left side :	++	++	++	++	++	++	++/+++

  - Genito-anus: no tenderness or palpable mass lesion or rectal shelf, normal external genitalia, normal anal tone, brown stool

CRS CONDITION

  -
    - Lung

手術日期及方法

- 手術日期及方法20210616 Low anterior resection with colo-anal anastomosis Ileostomy

住院治療經過

- Under the impression of s/p robotic-assisted LAR with suspect abscess formation over rectal anastomosis site, the patient was admitted for CT-guided drainage. PCD tube of presacral abscess was inserted on 2021/06/04. The drain was red initially and switched to yellowish, turbid discharge. The patient still complained of intermittent pain over puncture site and rectal region. Analgesic agent was added. We arranged colonscope on 6/9, which showed dehiscence over previous anastomosis site, compatible with image findings. We explained the current condition and re-do surgery for bowel re-anastomosis. The patient and his wife could understand. The operation of redo Low anterior resection with colo-anal anastomosis + Ileostomy were performed on 2021/06/16. We arranged post-op wound care and pain control after surgery. He began to try blend II diet on 6/21. Some red-dark discharge from anus was noted on 6/22, subsided later. He still complained of wound pain but tolerable. During 6/23-6/29, we kept post-op care. We has recommended him to perform sitz bath since 6/25. Foley was successfully removed on 6/28. Under relatively stable condition, he was discharged with drain. Suggest OPD follow up.

合併症

一般檢查

- **檢驗項目 :WBC,RBC,HGB,HCT,MCV 檢體:Blood 報告日期 :1100628**

CBC:

WBC	8700	/µL	(M:3900-10600;F:3500-11000)
RBC	3.44	x10^6/µL	(M:4.5-5.9;F:4.0-5.2)
HGB	10.2	g/dL	(M:13.5-17.5;F:12-16)
HCT	31.6	%	(M:40-52;F:37-47)
MCV	91.9	fL	(80.0-100.0)
MCH	29.7	pg	(26.0-34.0)
MCHC	32.3	g/dL	(31.0-37.0)
RDW	14.6	%	(11.5-14.5)
ANC	7343	/uL	(1780~6040/uL)
ALC		/µL	(1000 -3400/uL)
MPV	9.4	fL	(9 -13 fL)

備註:

Remark ：

兒童血液參考值(參考來源：中兒雜誌：167-73.1988)：

Age	RBC(×10^6µL)	Hgb(g/dL)	Hct(%)	MCV(fl)	MCH(pg)	MCHC(g/dL)
≦1day	4.01-4.93	14.2-17.2	43.3-52.3	103.9-110.1	33.9-36.3	32.5-33.3
>lday-8day	3.99-4.98	14.0-17.4	41.0-51.4	97.4-106.7	33.3-36.1	32.9-34.9
9day-≦6M	2.89-4.83	9.6-12.8	27.3-37.2	74.9-98.8	25.5-33.7	32.9-35.6
>6M- 6yr	4.28-5.05	11.6-1 3.7	34.2-39.8	74.9-84.6	25.2-29.1	32.6-35.1

Age	WBC(µL)	Neutrophils(%)	Lymphocytes(%)	Monocytes(%)	Eosioophils(%)
≦1day	14300-24900	61.2- 79.6	12.9- 27.7	3.5-9.5	0-3.3
>lday-8day	5200-13400	32.6- 70.7	16.8- 48.1	5.2-1 9.6	1-6.1
9day-≦1yr	5.00-12000	9.4-30.4	55.6-82.6	1.2-11.7	0.1-7.3
>lyr-≦4yr	6700-11800	13.9-49.5	44.7-81.6	1.3-7.2	0-4.3
>4yr-6yr	6000-10400	27.8- 57.6	34.4-62.8	2-7.6	0-6.8

檢驗項目 :Differential Count(D.C),Platelet 檢體:Blood 報告日期 :1100628

DC: SEG. % (40-74) BAND % (0-5) LYM 5.6 % (19-48)
MONO. 5.3 % (3.4-9) EOSINO. 4.4 % (0-7) BASO. 0.3 % (0-1.5)
META. % (0) MYELO. % (0) PROMYELO. % (0)
BLAST. % (0) GRAN. % Atypical Cell %
ATYP.LYM. % NEUT. 84.4 % (40-74)
NRBC/100WBC. 0.0
PLATELET COUNT. 488 x10^3/µL (150-400)

備註:

Remark ：

檢驗項目 :Bilirubin Total,GOT,GPT 檢體:Blood 報告日期 :1100628

AST(SGOT)	19	U/L	(8 - 38)
ALT(SGPT)	16	U/L	(M:10-50,F:10-35)
ALP		U/L	(50-190)
Total Bilirubin	0.41	mg/dL	(0.2-1.2)
Direct Bilirubin		mg/dL	(=0.2)(自106年10月20日起修訂Direct Bilirubin生物參考區間)
LDH		U/L	(120-240)
Uric Acid		mg/dL	(M:2.4-7.2, F:2.5-6.3)
Cholesterol		mg/dL	(200)
Triglyceride		mg/dL	(150)
FIB-4			(Metavir fibrosis stage F3 >= 3.25)

備註:

Remark ：

檢驗項目 :Albumin,BUN,Creatinine,Ca,Na,K 檢體:Blood 報告日期 :1100628

BUN	13	mg/dL	(5- 25)
Creatinine	0.78	mg/dL	(M:0.7-1.4 , F:0.5-0.9)
K	4.1	mEq/L	(3.5-5.3)
Na	141	mEq/L	(137-153)
Cl		mEq/L	(95-105)
Ca	8.8	mg/dL	(8.4-10.2)
Total Protein		g/dL	(6.0-8.0)
Albumin	3.0	g/dL	(3.5-5.0)

- eGFR 105.84 mL/min/1.73㎡ (Stage 1: >=90 ; Stage 2: 60-89 ; Stage 3: 30-59.9 ; Stage 4: 15-30 ; Stage 5: 15)

  備註:
  Remark ：

特殊檢查

病理報告

- 活體組織切片病理報告 手術日期：**110/06/16** 病理號：**S1019174**

  診斷醫師:王任卿 報告日期:110/06/22
  Pathologic diagnosis:
  Rectum, low anterior resection --- Status post CCRT and robotic LAR. Granulation tissue, suppurative inflammation with microabscess and stitch granuloma of mesocolon. No evidence of residual malignancy seen.
  Gross description:
  The specimen consists of a segment of colon, 14.5 cm in length and 3 cm in proximal cut end circumference, 4.5 cm in distal circumference, and perirectal soft tissue. On cut, focal rough serosal surface is noted near the distal cut end, about 5x2 cm. A separated firm nodule is noted at mesocolon. There is no mass lesion noted at mucosa. The both cut ends are unremarkable grossly. Representative part for section: A) proximal cut end B) distal cut end C-E, G-M) serosal lesion F) separated mesocolic lesion.
  #T-59600_2 #M-42000_2 0 1105 SP0000

其它

出院時情況

- 3.改門診治療

出院指示

藥名	劑量	劑量單位	用法	頻次	數量	數量單位	現況	天數	單位
(□)Takepron OD tab 30mg	1	TAB	PO	QDAC	5	TAB	TH	5	天
Anxiedin tab 0.5mg	1	TAB	PO	QN	5	TAB	TH	5	天
5mg Eliquis tab	1	TAB	PO	BIDPC	10	TAB	TH	5	天
25mg Utapine tab	0.5	TAB	PO	BID	5	TAB	TH	5	天
Voren-K tab 25mg	1	TAB	PO	TID	15	TAB	TH	5	天
BethanechoL tab 25mg-JS	1	TAB	PO	TIDAC	15	TAB	TH	5	天
1.25mg Bisoprolol tab	1	TAB	PO	QD	5	TAB	TH	5	天
OCAS Harnalidge 0.4 mg	1	TAB	PO	QDAC	5	TAB	TH	5	天
CefaDROxil-500 cap	1	CAP	PO	Q12H	10	CAP	TH	5	天

出院計劃

主治醫師 2222H 林俊余 / 住院醫師 2343L 張嘉育

正式摘要(電子簽章：**000000219845109)**

W72 / W72

doctorRank:B
